# Supplementary material for: Chronic Exposure to Chlorpyrifos Damages Thyroid Activity and Imbalances Hepatic Thyroid Hormones Signaling and Glucose Metabolism: Dependency of T3-FOXO1 Axis by Hyperglycemia
Source: Int J Mol Sci. 2023 May 31;24(11):9582. doi: 10.3390/ijms24119582 (PMC10253621; doi:10.3390/ijms24119582)
Supplement: Supplementary file 1 [file ijms-24-09582-s001.zip › ijms-2289184-supplementary.pdf]

Supplementary Materials

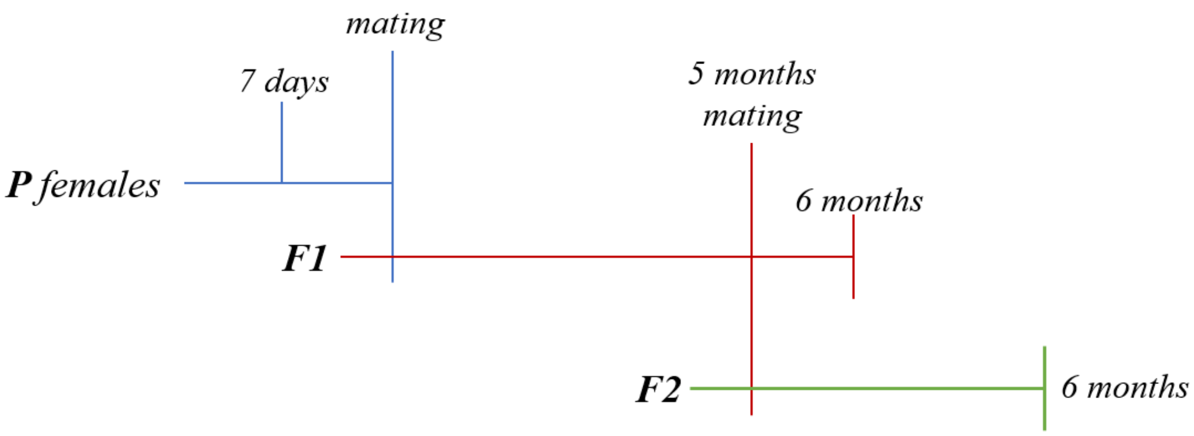

Supplementary Figure S1. Experimental plan. CD1 dams parental generation (P) were treated from 7 days before mating by feeding with food added with to CPF 0.1, 1, 10 mg/kg/die to generate F1 generation. These were exposed by the mother, lactation and subsequently exposed by feeding as above. F1 mice (5 months old) were mated to create F2 generation exposed as F1. F1 and F2 mice were sacrificed after 6 months exposure.

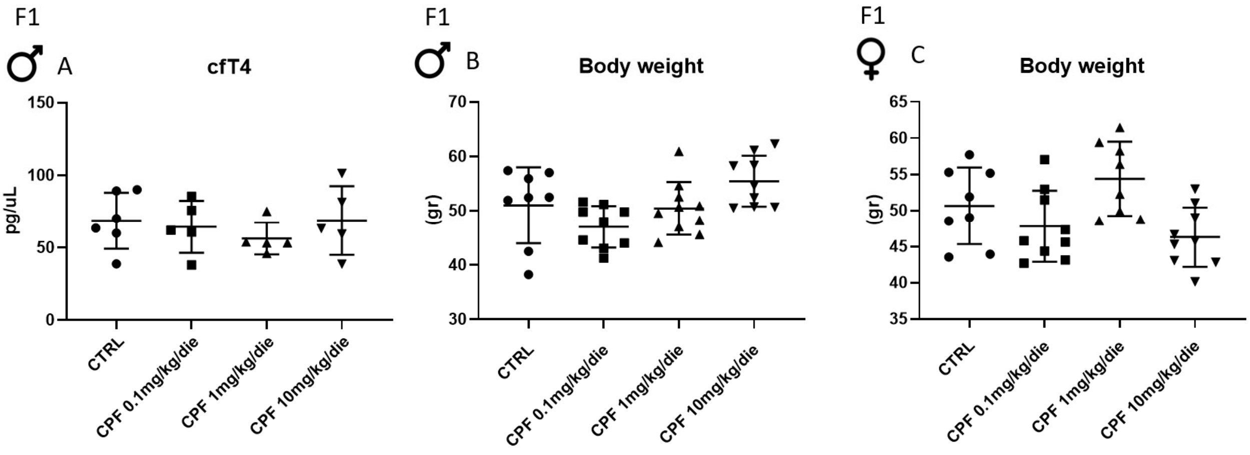

Supplementary Figure S2. Analysis of cfT4 content and metabolic parameters. (**A**) Serum levels of cfT4 in F1 6 months male mice. (**B**,**C**) Body weight of F1 6 months male and female treated with CPF 0.1, 1, and 10 mg/kg/die.

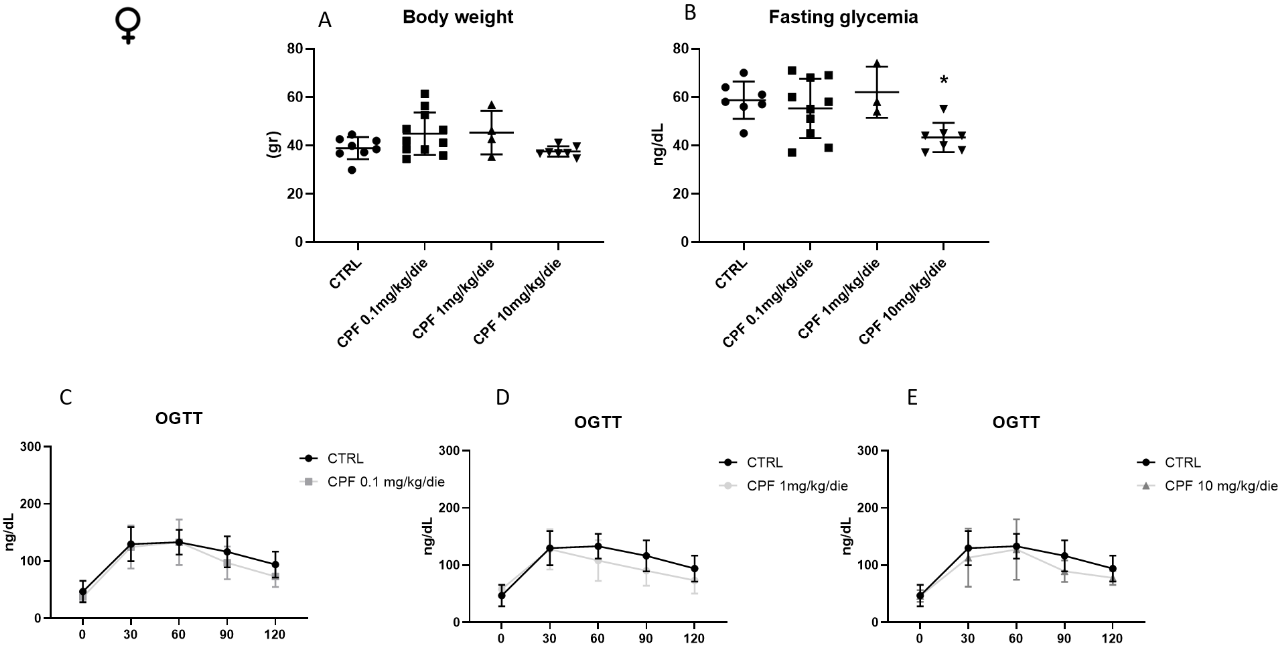

Supplementary Figure S3. Analysis of metabolic parameters in 6 months female mice. (**A**) Body weight of 6 months F2 females treated with CPF 0.1, 1, and 10 mg/kg/die. (**B**) Serum glucose levels after 16 hours of fasting were determined in mice treated with CPF 0.1, 1, and 10 mg/kg/die as described in M&M section. One-way ANOVA * $p < 0.05$ vs. CTRL ($n = 5$). (**C–E**) Plasma glucose concentrations ($n = 5$) during the intraperitoneal glucose tolerance test (OGTT) (2 g/kg) at 0, 30, 60, 90, 120 min in mice treated with CPF 0.1, 1 and 10 mg/kg/die. Data are reported as mean ± SD.

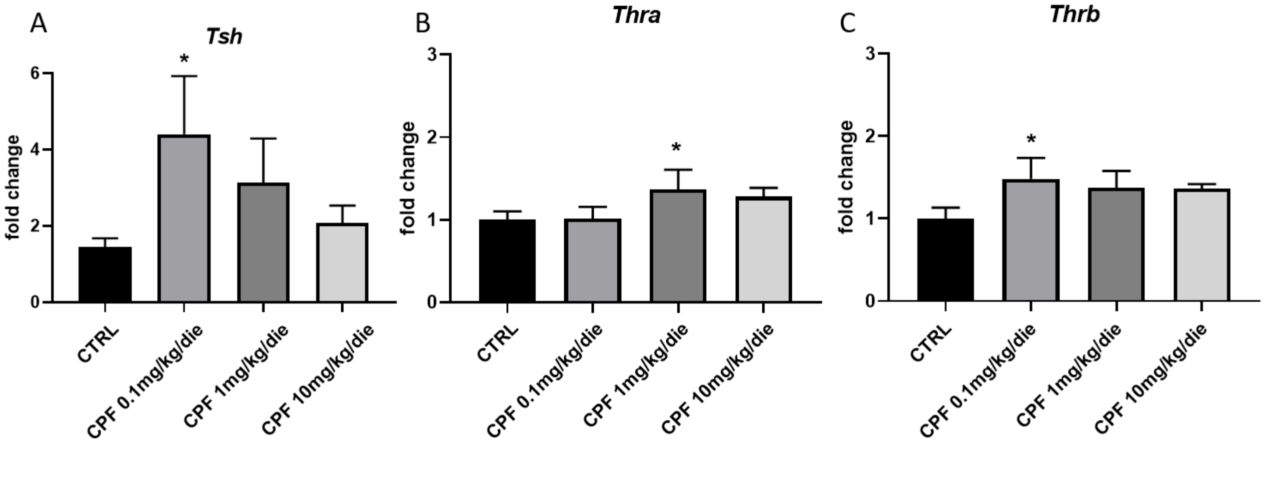

Supplementary Figure S4. Pituitary *Tsh* gene expression and hepatic expression of thyroid hormones receptors Thrs. (**A–C**). Pituitary expression levels of *Tsh* (**A**) measured by RTqPCR. Data were normalized to the values obtained for CTRL animals (set as 1) and shown as mean ± SD. At least 3 samples/group were used. Levels of *ThRa* (**B**), *ThRb* (**C**) by RTqPCR. Data were normalized to the values obtained for CTRL animals (set as 1) and shown as mean ± SD. At least 3 samples/group were used. One-way ANOVA * $p < 0.05$ vs. CTRL.

Table S1.

Table S1. List of primers used for gene expression analysis by quantitative Real Time PCR. *mm = Mus musculus.*

**Gene name**	**Species**	**Forward 5′-3′**	**Reverse 5′-3′**
Acc1	*mm*	ttcactgtggcttctccagc	gaccaccgacggatagatcg
b-actin	*mm*	gtcgagtcgcgtccacc	gtcatccatggcgaactggt
Dio1	*mm*	ccaccttcttcagcatcc	agtcatctacgagtctcttg
Fasn	*mm*	gactcggctactgacacgac	cgagttgagctgggttaggg
G6pase	*mm*	ctcactttccccaccaggtc	gaatccaagcgcgaaacca
Glut2	*mm*	ccagtacattgcggacttcctt	ctttcctttggtttctggaacttt
Pck1	*mm*	aggcagtgaggaagttcgtg	agccagccaacagttgtcat
ThRa	*mm*	gccattggaaacagaggcga	atctggtcttcgcaaggca
ThRb	*mm*	aaagtcacccgcaaccagt	atttctgtagctcttcccgcc
